# Awareness and safe practices of Hepatitis-B and C prevention and transmission among workers of women beauty salons

**DOI:** 10.12669/pjms.38.8.6166

**Published:** 2022

**Authors:** Hafiza Hifza Bashir, Lubna Kamani, Madiha Usman, Kajol Kishwar

**Affiliations:** 1 Hafiza Hifza Bashir, Liaquat National Hospital, Karachi, Pakistan; 2 Lubna Kamani, Liaquat National Hospital, Karachi, Pakistan; 3 Madiha Usman, Liaquat National Hospital, Karachi, Pakistan; 4 Kajol Kishwar, Liaquat National Hospital, Karachi, Pakistan

**Keywords:** Chronic hepatitis, Beauty salon, Hepatitis-B and C, Safe practice, Sterilization

## Abstract

**Background & Objectives:**

Hepatitis-B and C is currently a major health problem all over the world including Pakistan. All beauty treatments including manicures are used by many people and can be a risk factor because of sharing of contaminated instruments. Proper sterilization needs to be achieved by an autoclave. Our study was conducted to know the awareness and safe practices of Hepatitis-B and C prevention and transmission by beauty salon workers in Karachi.

**Methods:**

This was a cross-sectional descriptive study performed from February 2021- July 2021 among workers of women’s beauty salons across Karachi. Validated questioners were distributed and were filled in the presence of research worker. Data was compiled and analyzed using SPSS version 22. Workers who scored ≥ 70% were considered to have adequate knowledge.

**Results:**

Our results showed that out of 261 participants, 240 (92.3%) were females. 49(18.8%) had adequate knowledge about hepatitis-B, 63(24.1%) had adequate knowledge about hepatitis-C. 111(42.5%) had adequate practices. According to the independent T test, there was statistically significant relationship between family history of hepatitis-B and knowledge of Hepatitis-B (p=0.022), hepatitis-B vaccination and knowledge of Hepatitis-B (p=0.006). We also found significant relationship between family history of hepatitis-C and knowledge of hepatitis-C (p=0.019), also between previous blood test performed for hepatitis antibodies and knowledge about hepatitis-B and C. On Uni-Variate logistic regression we found that males participants are less likely to have adequate Hepatitis-C knowledge in comparison of female participants (OR=0.152). We also found that participants who have Hepatitis-B family history, have more likely to have adequate Hepatitis-C knowledge (OR=1.874) and males participants are less likely to have adequate Hepatitis-B knowledge in comparison of female participants (OR=0.212). Only 45(17.2%) workers were fully vaccinated with Hepatitis-B and 126(48.3%) had knowledge of adequate sterilization technique of equipment’s.

**Conclusion:**

This study showed that overall awareness among workers of women beauty salon in Karachi about Hepatitis-B and C is inadequate with low vaccination rates. There is dire need to organize awareness programs with mass vaccination campaigns for safe practices and to curb viral transmission.

## INTRODUCTION

Hepatitis-B and Hepatitis-C are viral infections and the major health problems because of its association with the high rate of chronicity and its progression to decompensation and liver cancer, approximately 240 million people are chronically infected with HBV and 130–150 million with HCV all over the world with a 3-4% carrier rate of hepatitis B.^1^,^2^ In Pakistan, seven to nine million people are living with HBV While about 10 million people are living with HCV in Pakistan.^3^ Due to larger Pakistani population (165 million) and increased rates of infection it is considered to be the worst afflicted nation.^4^ Prevalence of these diseases in barber shops in Pakistan has been reported to be 34% to 49%.^5^ This infection has enormously increased in last two decades in Pakistan.^6^

Hepatitis-B infection is a health-care problem that needs immunization program and infection control services, awareness programs about injection safety and blood safety.^7^ Hepatitis-C-related liver cirrhosis is the seventh highest cause of mortality around the world hence elimination of viral hepatitis should be the first priority.^8^ Viral hepatitis eradication is not possible in most underdeveloped countries and middle-income countries. In contrast to HBV, there is no vaccine available for Hepatitis-C so it is clear that the WHO goals for incidence reduction will not be easy to achieve in case of Hepatitis-C.^9^ Moreover, the impact of Covid-19 pandemic on hepatitis elimination is detrimental and leaving our efforts further behind. After infection with Hepatitis-B or C some people may develop acute hepatitis. But in some patients it goes un-noticed and becomes chronic hepatic infection while others get spontaneously resolved. If not treated timely viral Hepatitis-Can cause serious health complications. More than 20% who has chronic infection develop decompensated liver cirrhosis or liver cancer.^10^

Common routes of spread are intravenous drug use, blood transfusions, prick injuries among health care workers, sexual transmission and congenital transmission.^11^ All beauty treatments including manicures are used by many people and can be a risk factor because of sharing of contaminated sharp instruments these instruments are infected if not sterilized properly can lead to spread.^12^ Nail scissors, shared use of nail scissors, and duration of shared use play important roles in transmission of HBV.^13^ Therefore, extensive sterilization of therapeutic and beauty instruments to prevent spread of Hepatitis-B and C is important particularly because these viruses are not easily removed by drying, using simple detergents.^14^

This virus can live up to seven days outside body, still being highly resistant. Contaminated instruments are real threat for both, client and salon. Ideally sterilization of every tool should be done by autoclave, in beauty salons, the hot air oven that provides dry heat sterilization is widely used. This type of sterilization need prolong exposure time at high temperatures, so that the penetration and heat distribution are equal which is more effective way of sterilization^15^ Sterilization should only be done after removing dust with detergent soap and water would decrease amount of microorganisms.^16^ The purpose of our study was to access the awareness and safe practices of Hepatitis-B and C prevention and transmission among workers of women beauty salons in Karachi.

## METHODS

We conducted cross-sectional descriptive study from February – July 2021 among workers of women beauty salons of Karachi. Ethical approval of our study was received from Ethical committee of Liaquat National Hospital Karachi. (Reference # 0594-2020).

Those who were willing to participate in the study and involved in procedures with experience of more than six months were included after informed consent. The calculated sample size of our study was 261 workers after taking frequency of intermediate knowledge in salon workers, P=57.6% (32) by using marginal of error=6% We selected beauty salons randomly by convenient sampling. Pre testing of the questionnaire (59 questions) was performed and later distributed among salon workers and it was filled in the presence of a research worker. Data was compiled and analyzed using statistical package for social sciences (SPSS) version 22. Workers who scored ≥ 70% were considered to have adequate knowledge.

**Fig.1:**
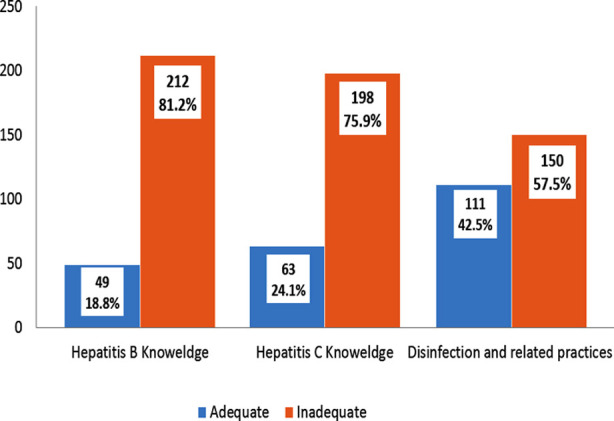
Mean of Hepatitis-B and Hepatitis-C knowledge and adequate practices.

Mean and standard deviation was calculated for quantitative variable. Frequencies and percentages were calculated for qualitative variable. Post-stratification chi square test was applied taking p value of <0.05 as statistically significant.

## RESULTS

Total 261 participants were included. There were predominantly write number also (92.3%) female participants. Most of the participants were of 20 years with a range of 15-25 (Table-I). About 49(18.8%) had adequate knowledge about Hepatitis-B, 63(24.1%) had adequate knowledge about Hepatitis-C, 111(42.5%) had adequate practices, 45(17.2%) workers were vaccinated. Hepatitis-B and C knowledge was assessed on the basis of questions as shown in Table II-IV.

**Table-I:** Basic Demographic Details.

	n(%)
***Gender***	
Male	20(7.7)
Female	241(92.3)
***Education***	
No education	8(3.1)
Primary	20(7.7)
Middle(matriculation)	84(32.2)
Secondary	98(37.5)
Graduation	42(16.1)
Masters	9(3.4)
***Age Group***	
15-25	124(47.5)
26-35	84(32.2)
36-45	39(14.9)
>45	14(5.4)
***Working Experience in Saloon***	
<1 year	10(3.8)
1-5 years	101(38.7)
5-10years	80(30.7)
10 -15years	33(12.6)
>15 years	37(14.2)
***Hepatitis-B Family History***	
Yes	14(5.4)
No	218(83.5)
No idea	29(11.1)
***Hepatitis-C Family History***	
Yes	30(11.5)
No	199(76.2)
No idea	32(12.3)
***Hepatitis-B Vaccination done***	
Yes	45(17.2)
No	216(82.8)
***Blood Test done for hepatitis antibodies***	
Yes	44(16.9)

**Table-II:** Mean of Hepatitis-B and Hepatitis-C knowledge and adequate practices:

	Mean±SD	Range
Correct Answers regarding Hepatitis-B Knowledge	8.44±3.50	0 to 15
Hepatitis-B Knowledge Percentage	49.68±20.62	0 to 88
Correct Answers regarding Hepatitis-C Knowledge	8.70±3.63	0 to 16
Hepatitis-C Knowledge Percentage	51.20±21.33	0 to 94
Correct Answers regarding Disinfection and related practices	10.75±2.60	2 to 16
Disinfection and related practices Percentage	67.21±16.26	12.5 to 100

**Table-III:** Responses on questions regarding Hepatitis-B knowledge.

	Yes	No	No idea
Is Hepatitis-B be transmitted through kissing?	62(23.8)	98(37.5)	101(38.7)
Is Hepatitis-B be spread by using same toilet seat of patients?	92(35.2)	96(36.8)	73(28)
Is Hepatitis-B be spread by insect bit?	73(28)	112(42.9)	76(29.1)
Is Hepatitis-B be appear in a child of affected mother?	101(38.7)	112(42.9)	48(18.4)
Is Hepatitis-B run in families?	101(38.7)	112(42.9)	48(18.4)
Is Hepatitis-B be transmitted through sexual intercourse?	140(53.6)	49(18.8)	72(27.6)
Is Hepatitis-B be transmitted by communicating with the patient?	40(15.3)	166(63.6)	55(21.1)
Can Hepatitis-B be transmitted through nail clippers?	163(62.5)	53(20.3)	45(17.2)
Can Hepatitis-B be transmitted through embracing and hand shaking?	56(21.5)	142(54.4)	63(24.1)
Can Hepatitis-B be spread by having the food with patient and using same utensils of the patients?	100(38.3)	106(40.6)	55(21.1)
Should Hepatitis-B patients be completely isolated in the family?	45(17.2)	169(64.8)	47(18)
Can Hepatitis-B be transmitted through sharing a towel?	118(45.2)	86(33)	57(21.8)
Can Hepatitis-B be spread by using same razors and shaving machines of another person?	165(63.2)	38(14.6)	58(22.2)
Blood as vehicle for Hepatitis-B	166(63.6)	21(8)	74(28.4)
Is HBV an occupational risk	127(48.7)	58(22.2)	76(29.1)
Is there vaccine available for Hepatitis-B in Pakistan?	141(54)	37(14.2)	83(31.8)
Is Hepatitis-B treatable?	162(62.1)	40(15.3)	59(22.6)

**Table-IV:** Responses on questions regarding Hepatitis-C knowledge.

	n (%)		

	Yes	No	No idea
Is Hepatitis-C transmissible by kissing?	64(24.5)	98(37.5)	99(37.9)
Is Hepatitis-C transmissible by using same toilet seats of patients?	95(36.4)	98(37.5)	99(37.9)
Is Hepatitis-C transmissible through insect bit?	78(29.9)	110(42.1)	73(28)
Is Hepatitis-C can be acquired by child of infected mother?	161(61.7)	37(14.2)	63(24.1)
Does Hepatitis-C run in families?	98(37.5)	110(42.1)	53(20.3)
Is Hepatitis-C be transmitted through sexual intercourse?	135(51.7)	56(21.5)	70(26.8)
Is Hepatitis-C transmissible by communication with the patients?	51(19.5)	158(60.5)	52(19.9)
Is Hepatitis-C transmissible through nail clippers?	153(58.6)	61(23.4)	47(18)
Is Hepatitis-C can spread through embracing and hand shaking?	58(22.2)	146(55.9)	57(21.8)
Is Hepatitis-C spread by having the food and using same utensils of patients?	101(38.7)	104(39.8)	56(21.5)
Should Hepatitis-C patients be left out alone?	48(18.4)	157(60.2)	56(21.5)
Can Hepatitis-C be spread by using same towel of another person?	116(44.4)	86(33)	59(22.6)
Can Hepatitis-C be spread by using same razors and shaving machines of another person?	162(62.1)	41(15.7)	58(22.2)
Blood as vehicle for Hepatitis-C	165(63.2)	21(8)	75(28.7)
Is HCV an occupational risk	128(49)	58(22.2)	75(28.7)
Does vaccine is available for Hepatitis-C in Pakistan?	141(54)	37(14.2)	83(31.8)
Is Hepatitis-C treatable?	162(62.1)	40(15.3)	59(22.6)

There was a significant association of adequate Hepatitis-B knowledge with Hepatitis-B family history (p=0.022) and Hepatitis-B vaccination (p=0.006). On Uni-Variate logistic regression we found that males participants are less likely to have adequate Hepatitis-B knowledge in comparison to their female participants (OR=0.212), participants who have Hepatitis-B family history were more likely to have adequate Hepatitis-B knowledge in comparison with no family history (OR=3.238).

There was significant association of adequate Hepatitis-C knowledge with Hepatitis-C family history (p=0.019) and Hepatitis-C vaccination (p=0.044). On Uni-Variate logistic regression analysis we found that males participants are less likely to have adequate Hepatitis-C knowledge in comparison to female participants (OR=0.152) also participants who have a family history of Hepatitis-C have adequate knowledge of Hepatitis-C (OR=1.874).

There was no significant association of disinfection and related practices with gender (p=0.100), education (p=0.520), age group (p=0.126), working experience (p=0.126), Hepatitis-B family history (p=0.868), Hepatitis-C family history (p=0.611), Hepatitis-B vaccination (p=0.479) and blood test done for hepatitis antibodies (p=0.055). By Uni-Variate logistic regression analysis we found that males participants were more likely to have adequate disinfection and related practice. (OR=2.152).

## DISCUSSION

Hepatitis-B and C are transmitted by infected blood and virus can be highly infectious even if remains outside the body for a week, which means that during beauty procedures even minor bleeding can cause entrance of pathogenic organism. Therefore, it is emphasized that every instrument which is being utilized in beauty salons should be sterilized extensively in autoclave or sterilizer box, as clients usually do not carry their personal kits and there are high chances of viral transmission during minor cuts or abrasions.^12^ Autoclaving is the best way to kill microorganisms, although process takes a longer time and also not good for electrical instruments, chemical disinfectants need to be used with caution and are effective in killing microbes or slowing their growth^17^ Saloon workers are vulnerable to many occupational health risks. Long working hours, prolong standing with poor posture with mechanical joint loads, and acquiring Hepatitis-B and C are major risks for barbers and saloon workers.^18^ Lack of awareness, and knowledge, unavailability of space and equipment and rapid turnover of customers are few reasons for improper sterilization. Instead of autoclave, most salons have hot air oven that provides dry heat sterilization. This type of sterilization needs prolong exposure time at high temperatures, so that the penetration and heat distribution are equal but not more effective than autoclave.^19^

This is the first study to our knowledge which was conducted among women beauty salon workers in various city areas of Karachi as the previous studies have been done on Barbour shops. During our survey we also noticed that workers were not much knowledgeable but their hand hygiene practices were better and this could be the current pandemic situation of COVID-19.

In a study done by Waheed Y et al. in 2010 in Pakistan^17^ about barbers’ awareness 90.7% were aware about transmission of Hepatitis-B and C by blade sharing, 47.8% knew that Hepatitis-B vaccine is available and 9.8% were vaccinated against Hepatitis-B, 42.9% were thinking that Hepatitis-C vaccine is available. In our study 63.2%, 62.1% were aware that Hepatitis-B and C can be spread by razors respectively. Another 54.1% were aware that Hepatitis-B vaccine is available and only 17.2% were vaccinated against Hepatitis-B. 54% thought that Hepatitis-C vaccine is available. In another study done in Pakistan^18^ showed that only 46% of participants had knowledge about availability of Hepatitis-B vaccine and also being the part of EPI program while in our study 54.1% participants knew that Hepatitis-B vaccine is available and they also said that they are vaccinated as per EPI program.

In another study done in Pakistan by de Oliveira ACDS et al.^20^, the knowledge of Barber’ about hepatitis disease and transmission risk was so low and commonly reuse razors. In our study mostly workers were not using razors and they were also aware of the risk of transmission through razors. In a similar study carried out by Freeland et al.^21^ out of 715,46% of men, 27% of women were aware that hepatitis B is not transmitted by food prepared by an affected person, in our study among 262 workers, 38.3% knew that Hepatitis-B is not transmissible through food and utensils of the patients, 39.8% knew that Hepatitis-C is not transmissible by food and utensils of the patient. In another study done in Peshawar, Pakistan^22^ 14.3% reported using gloves and 19.4% reported using only aprons. Another 20.4% of beauty therapists claimed sterilizing their instruments between clients, while in our study 68(26.1%) were sterilizing hair brushes in between customers and 237(90.8) were sterilizing manicure, pedicure kits after every use.

In another study done in Isfahan by Ataei B et al.^23^ among hairdressers about evaluation of awareness and practices of Hepatitis-B and C, 23.6% clients were carrying their personal cosmetic suit 5.7% were disinfecting manicure and pedicure kits in boiling water after every use, 72.9% were using fresh towels for each client. In our study 26.1% were carrying their personal cosmetic suit, 90.8% were disinfecting manicure and pedicure rasps in boiling water or sterilizer box, 91.6%were using fresh towels for each client which clearly shows that workers were practicing adequate, and the possible could be the current pandemic situation although overall awareness about Hepatitis-B and C was not adequate.

In our study 62.5%, 58.6% knew that Hepatitis-B and C are transmissible by nail clippers respectively and the results are in line with previous study done by Ataei B et al.^23^ which showed similar result of 61.1%. This study showed that overall awareness about Hepatitis-B and C is inadequate, although practices were better but overall knowledge of Hepatitis-B and C were lacking among salon workers. Additionally, there is need to focus on educating and spreading awareness through media campaigns, providing education and arranging mandatory courses for female salon workers Also clients should be encouraged about carrying their personal cosmetic manicure and pedicure kits as this can decrease chances of viral transmission to some extent.

On the other hand workers can also get exposed with contaminated body fluids from customers and it is imperative to get them fully vaccinated against Hepatitis-B, this should be mandatory for all the workers to be vaccinated as they are dealing with sharp instruments on daily basis Some of the workers also don’t consider that they are at occupational risk, hence more awareness programs for female salon workers need to be organized to increase their knowledge for safe practices, education of female salon workers about significance of sterilization of beauty instruments would definitely help in reducing the burden of community-acquired infection with blood-borne infections.

### Limitations of the study:

This study was conducted in Karachi salons only and might not be a true reflection of beauty salon practices in other parts of Pakistan.

## CONCLUSION

This study showed that overall awareness among workers of the women’s beauty salon in Karachi about Hepatitis-B and C is inadequate with low vaccination rates. There is a dire need to organize awareness programs with mass vaccination campaigns for safe practices and to curb viral transmission.

### Author’s Contributions:

**HHB:** Protocol writing, Data collection, Data analysis and editing of manuscript, accuracy and revision of manuscript.

**LK:** Conceptualization, Data analysis, Final drafting, Integrity of manuscript and critical revision of manuscript. She is also responsible for the integrity and accuracy of the study.

**MU:** Data collection and writing first draft of manuscript.

**KK:** Data entry, Data analysis and revising of manuscript.
